# Discovery of the first succulent bamboo (Poaceae, Bambusoideae) in a new genus from Laos’ karst areas, with a unique adaptation to seasonal drought

**DOI:** 10.3897/phytokeys.156.51636

**Published:** 2020-08-21

**Authors:** Thomas Haevermans, Dulce Mantuano, Meng-Yuan Zhou, Vichith Lamxay, Agathe Haevermans, Patrick Blanc, De-Zhu Li

**Affiliations:** 1 Institut de Systématique Évolution Biodiversité (ISYEB), Muséum national d’histoire naturelle, Centre national de la recherche scientifique, École Pratique des Hautes Études, Université des Antilles, Sorbonne Université. 45 rue Buffon, CP 50, 75005 Paris, France Sorbonne Université Paris France; 2 Germplasm Bank of Wild Species, Kunming Institute of Botany, Chinese Academy of Sciences, Kunming, Yunnan 650201, China Universidade Federal do Rio de Janeiro Rio de Janeiro Brazil; 3 Faculty of Natural Sciences, National University of Laos, Vientiane Capital, Lao PDR Kunming Institute of Botany, Chinese Academy of Sciences Kunming China; 4 Plant Ecophysiology Lab, Institute of Biology, Universidade Federal do Rio de Janeiro, Brazil National University of Laos Vientiane Lao People's Democratic Republic; 5 Centre national de la recherche scientifique (CNRS), 3 rue Michel-Ange, Paris, France Centre national de la recherche scientifique Paris France

**Keywords:** Bambusinae, desiccation tolerance, genetic resources, xerophyte

## Abstract

Lush jungle flagship species, woody bamboos (Poaceae–Bambusoideae) are famed for their synchronous flowering as well as the extensive “bamboo forests” some species can form in tropical or temperate environments. In portions of their natural distribution, Bambusoideae members developed various adaptations to seasonality in environmental parameters, such as frost or seasonal drought. A new taxon, *Laobambos calcareus*, described here, is extremely novel in showing the first documented case of succulence in bamboos, with its ability to seasonally vary the volume of its stem depending on the quantity of water stored. Anatomical studies presented in this paper document this specificity at the cellular level. Though no flowers or fruits are known yet, unique morphological characteristics along with an investigation of its phylogenetic affinities using molecular data show that this new taxon should belong to a new genus herein described.

## Introduction

Bamboos (Poaceae – Bambusoideae) presently natively occur in every part of the world except Europe and Antarctica (Vorontsova et al. 2016). They form a very large culturally, economically and ecologically important group of organisms by sustaining entire endangered ecosystems, or as a source of food or materials for humans. Bamboos are famed cultural elements, used for virtually every part of the daily life in Asia, from food, cooking utensils, garden ornaments to house building material (Wong 2004). Bamboos are immediately recognizable by their typical habit while showing countless variations on the same model, from species with large woody culms, to small shrubby understory woody or herbaceous plants. The diversification of the group led to unique morphologies and adaptations, such as fire-resistance, as found in the South American genera *Actinocladum* McClure ex Soderst. (Soderstrom 1981), or *Glaziophyton* Franch. Bamboos even comprise truly climbing plants such as the Asian genus *Dinochloa* Buse, or the Malagasy *Sokinochloa* (Dransfield 2016). Complete seasonal deciduousness has been described in some instances, such as in Madagascar bamboos, occurring in tropical xeric conditions (Camus 1925) or correlated to cold tolerance in the temperate *Arundinaria appalachiana* Triplett, Weakley & L.G. Clark (Triplett et al. 2006). Some Mesoamerican *Otatea* taxa occur in extremely dry areas with annual precipitations as low as 350 mm (Ruiz-Sanchez 2015). However, “succulence”, i.e. the capacity to store water in a specific organ whose volume varies, has never before been documented for a bamboo.

The new taxon described herein, presenting a combination of both complete seasonal deciduousness and stem succulence, was collected in a karstic massif in Khammouane province in central Laos, during a Radeau des Cimes (“Canopy Raft”) expedition (Opération Canopée 2015). The climate is tropical, receiving more than 2000 mm of precipitation per annum, and characterized by a strong seasonality with a marked dry season from October to April/May (with virtually no rainfall during 4 months from November to February) and a very wet season from June to September with a monthly average of 400 mm of precipitation, and 25–30 rainy days monthly (World Weather Online 2020).

**Figure 1.**
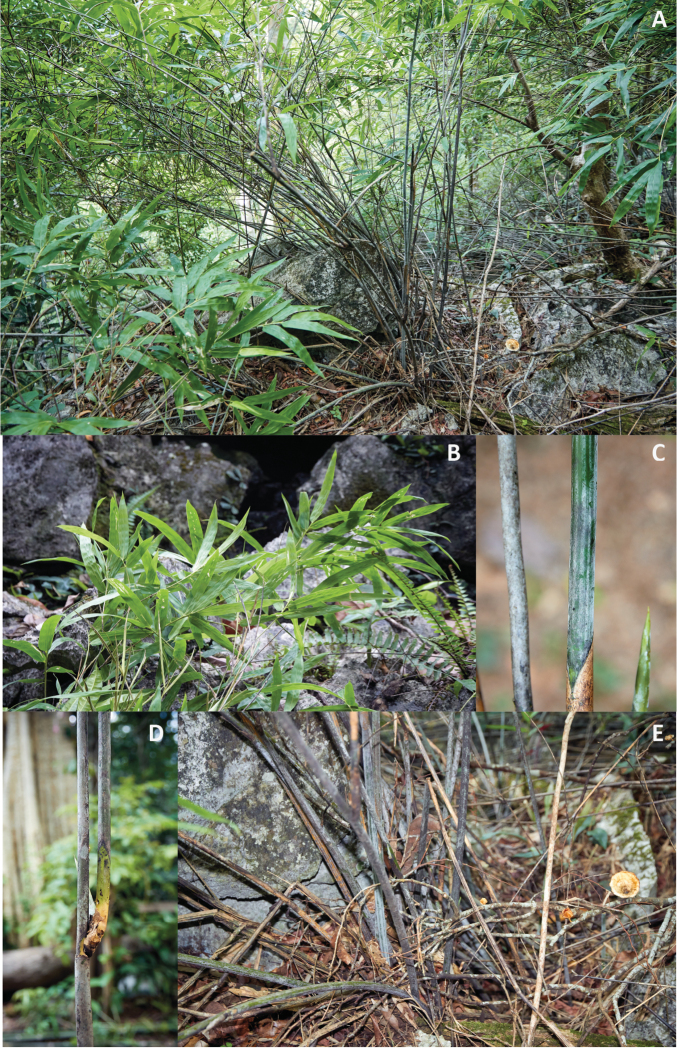
*Laobambos calcareus*. **A** Adult plant in habitat at the type locality during rainy season **B** detail of foliage leaves **C** detail of the waxy marks on fresh inflated culms, revealing the ridges present on the dried-state culm during the dormant period **D** detail of a node and a branch complement **E** view of base of the clump showing the greenish inflated fresh live culms, along with ridged dehydrated brown dead culms. (Photo credit Thomas Haevermans 05/2012).

This plant was first spotted by the Canopy raft scouting expedition, during the dry season in January while the plants were leafless and deflated due to local extreme xeric conditions. In dormant state, the “deflated” aspect of the culms observed by the scouting expedition could easily be mistaken by a non-specialist as belonging to the orchid genus *Dendrobium* Sw., emphasizing the very peculiar wrinkled aspect of its culms and main branches when dormant. When subsequently visiting the same locality during the beginning of the rainy season, while the plants were in full leaf and the culms “inflated”, its “bambooness” was obvious in exhibiting typical architectural traits (Fig. 1). However, no fertile plants were located and only sterile specimens have been collected and documented. Several botanists later visited the area at various seasons to complement observations of seasonally expressed traits, but failed to find a fertile specimen. The authors also failed to locate any other specimens matching our new taxon in large international collections representative of the area visited.

While bamboo species descriptions based on sterile material only are generally avoided, there do, however, exist precedents in the literature such as *Oldeania ibityensis* (A.Camus) D.Z.Li, Y.X.Zhang & Haev. (Zhang et al. 2017), originally described as *Arundinaria ibityensis* A.Camus, based merely on sterile syntypes from Madagascar (Camus 1960), but supported by recent molecular evidence. *Aulonemia nitida* Judz. described from sterile material, from the Pakaraima mountains in Guyana, South America, is another example of description based on non-fertile material (Judziewicz 2005).

The present paper is thus an exception in describing a new bamboo species, in a new genus, based solely on macro-morphological and anatomical studies of sterile material (herbarium specimens and field observations) and DNA sequence comparison.

## Methods

Specimens were collected in Laos according to national and international regulations, and dried on-site with hot air; silica gel preserved leaf fragments were prepared during collection for subsequent DNA sequencing for use in comparative studies. Original specimens were deposited in Laotian national facilities for permanent storage, and duplicates were distributed to collaborating institutes for further reference and dissemination of the results. Drawings illustrating the description were prepared from the specimens.

Anatomical preparations of the leaves and the culms were performed from dry herbarium specimens. Freehand sections of subsequently rehydrated culms were done (without chemical treatments) to understand the distribution of the water storage elements in the plant. Additionally, leaf and culm sections from dried herbarium samples were remounted with heated 3% aqueous KOH, fixed with FAA 70%, sectioned with a Ranvier microtome, stained with Astra blue and Safranin red, and finally mounted in glycerinated water, to better describe the anatomical structure.

**Supplementary video file.** Rehydration of a dead dried stem section of *Laobambos calcareus* with distilled water. The stem section was 1.5–2.0 mm thick. Scale marks represent 1 mm. The real-time video lasts for 1:13min. The video is available from Figshare https://doi.org/10.6084/m9.figshare.11919003 (video credit Dulce Mantuano).

## Results

**Figure 2.**
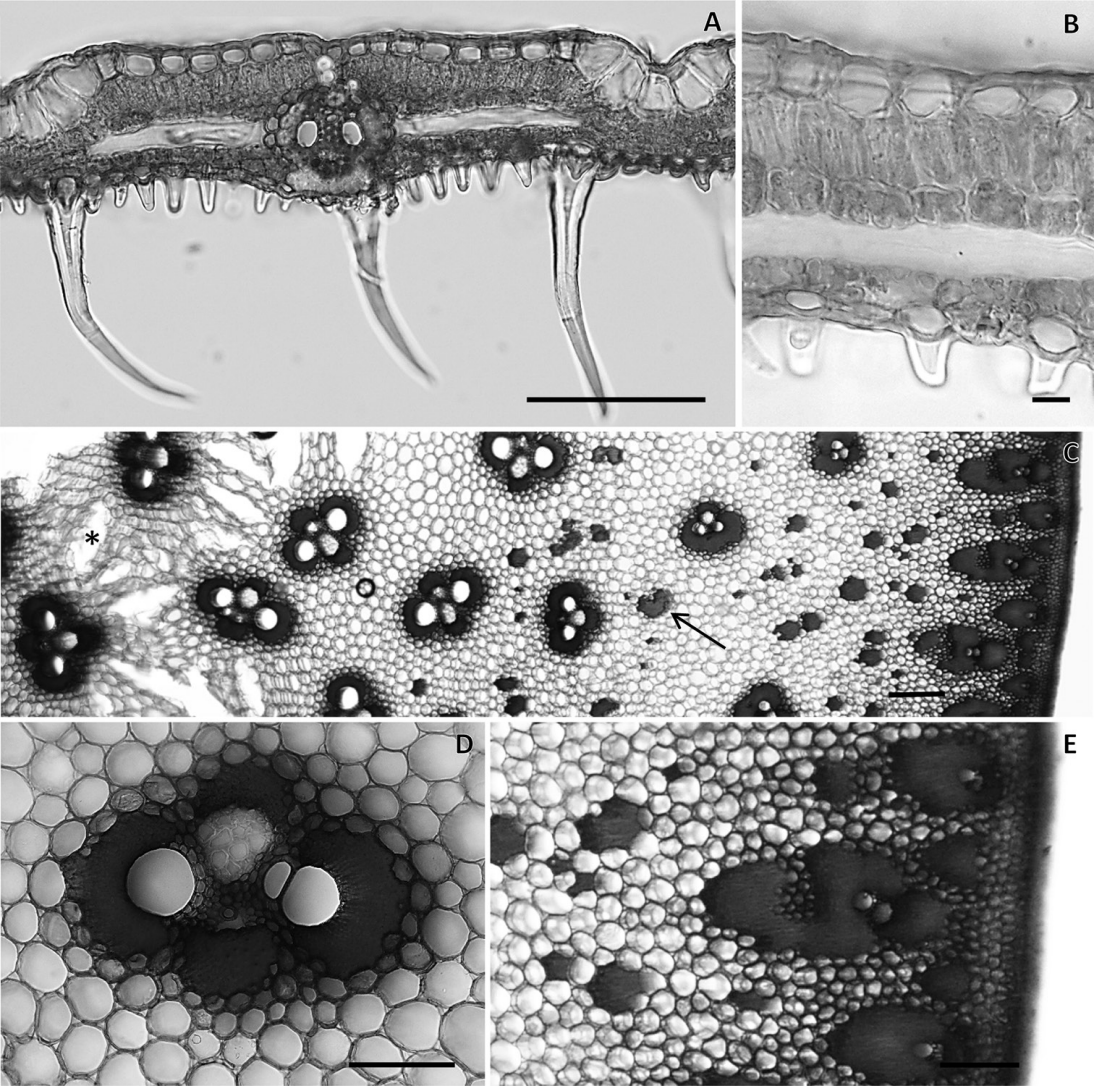
Transverse sections of leaf and culm of *Laobambos calcareus*. **A** Transverse section of a mature foliage leaf **B** mesophyll showing chlorenchyma with arm cells, fusoid cells and abaxial epidermis with papillae and trichomes **C** culm transverse section showing a medullary region (left side) with typical vascular bundles and cavities (asterisk) and a cortical region (right side) with isolated fiber bundles (arrow) **D** central vascular bundle in detail **E** cortical region in detail. Scale bars: 100 µm (**A, D, E**); 10 µm (**B**); 200 µm (**C**). (Photo credit Dulce Mantuano).

**Figure 3.**
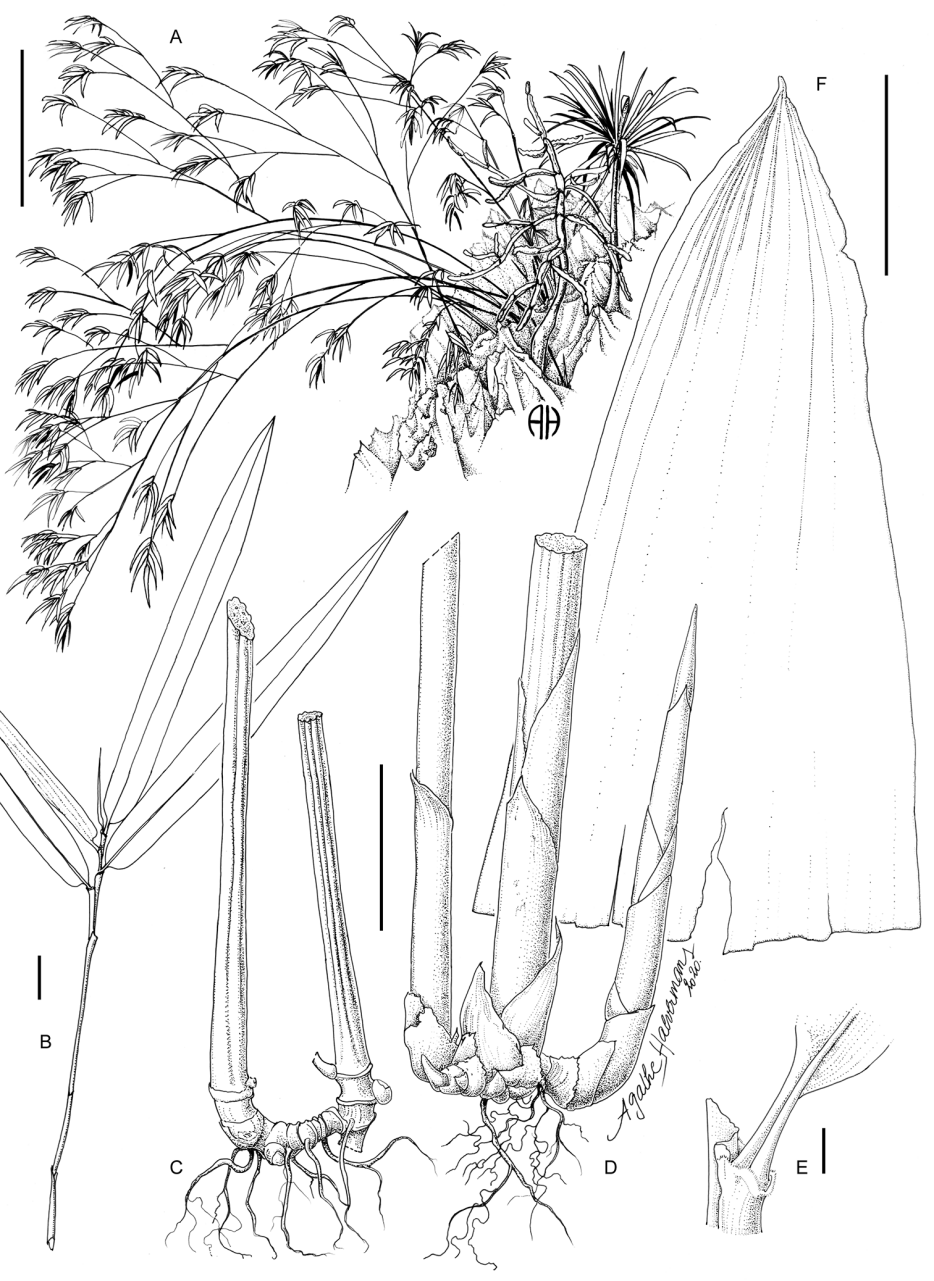
*Laobambos calcareus*. **A** Plant in habitat, growing in karst crevices typically along with *Euphorbia antiquorum* L. and *Dracaena cochinchinensis* (Lour.) S.C.Chen **B** leaf complement **C** plant in dormant state, rhizome leaves removed to show the structure **D** rhizome with year+1 shoot cross-section, and young shoots **E** petiole insertion with inner and outer ligules (view from below) **F** culm sheath, ventral view. Scale bars: 1 m (**A**); 2 cm (**B**); 5 cm (**C, D**); 2 mm (**E**); 1 cm (**F**). (Illustration credit Agathe Haevermans).

Figure 2A, B show that the leaf blades are not succulent and are essentially not distinct from typical bamboos leaves. The transverse section of a mature leaf (Fig. 2A) shows an adaxial epidermis presenting bulliform cells, chlorenchyma with two abaxial layers with arm cells, fusoid cells in the middle, one adaxial layer of arm cells, and an abaxial epidermis with papillae and trichomes. The stem shrinks in diameter during the dry season, forming grooves in the outer surface, as can be also be seen on herbarium specimens. The absolute water content of the mature culm was 1.63 g.g^-1^. The rhizome (extremely reduced in proportion to the plant) does not show the typical succulence and shrinkage wrinkles of the culm when dry (Fig. 3G, H).

The culm and branches are solid throughout, presenting numerous vascular bundles and isolated non-vascular fiber bundles embedded in the parenchymatous ground tissue. Cavities can be found in the pith region (Fig. 2C). Subepidermal parenchyma is present. Vascular bundles did not present a transition between central and peripheral morphologies. Vascular bundles in the central region presented four discontinuous sclerenchymatic fiber sheaths associated with two metaxylem vessels, phloem, and intercellular space (Fig. 2D). Vascular bundles in the peripheral region are radially oriented, protoxylem and metaxylem are surrounded by sclerenchyma, and phloem can be absent (Fig. 2E).

## Discussion

**Figure 4.**
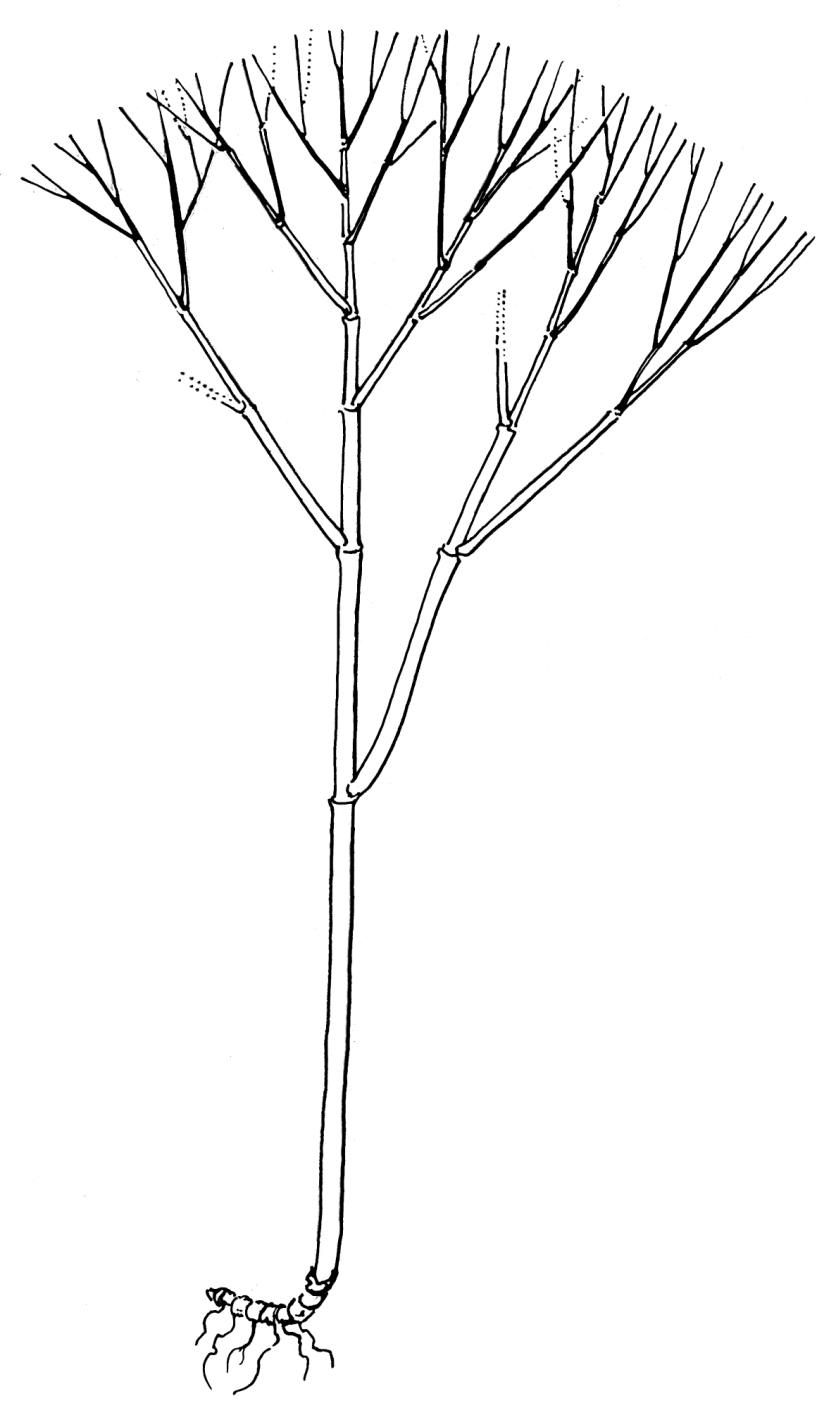
Scheme detailing *Laobambos calcareus* culm architecture illustrating the much elongated first internode, alternate branching with branch diameter matching the diameter of adjacent culm segment, and length of branches not exceeding the total length of the culm. (Illustration credit Agathe Haevermans).

This unique bamboo, to be named *Laobambos calcareus*, possesses solid succulent culms bearing single-branched branch-complements, culm leaves that are persistent, coriaceous throughout, and devoid of a blade, developed auricles, or oral setae. Its unequal culm-nodes pattern and branching architecture (Figs 4; 1D) is very unusual for a Paleotropical bamboo, and though unrelated to, reminds of some Neotropical Arthrostylidiinae possessing a first elongated internode followed by reduced ones (*Arthrostylidium schomburgkii* Munro or *Glaziophyton*) as documented in Tyrrell et al. (2012). The genus *Myriocladus* Swallen, also in this Neotropical Bambuseae group, expresses a pattern of internodes heterogeneity similar in some ways to *Laobambos*, but the two differ otherwise in terms of their respective architectures (Afonso et al. 2019).

The succulence of the culm, i.e. its capacity to store water by modifying its diameter, evident from macromorphological observation, is further informed by an anatomical cross-section suggesting the capacity for the bamboo to store water in zones distributed throughout the solid culm. Measured succulence (absolute water content) for mature culms is higher than previously reported for young, one year-old bamboo shoots; which usually varies between 1.2–1.3 g.g^-1^ (Rao 1985). Succulence of the culm is mainly due to the water-holding capacity of parenchyma cells, as no specific water-storage cells were identified. Surprisingly, a rapid rehydration could be observed even when the tissue is dead. A video in real time is provided as supplementary online material to show the speed at which a culm cross-section from a herbarium specimen can take water in. The ability to rapidly absorb water is probably linked to an adaptation to water pulses in the karstic rock habitat, desiccation tolerance (DT) being adaptively optimal for plants growing on substrates impenetrable to roots or experiencing seasonally dry conditions (Proctor and Tuba 2002). Variation in moisture content of different parts of culms were found to be clearly associated with the proportion of parenchyma cells in the tissue system (Liese and Grover 1961). Our anatomical findings corroborate this observation, although the culm structure of *Laobambos calcareus* does not show, as is typical of bamboos, a dense vascular bundle transition layer (Grosser and Liese 1971), but rather sparsely distributed vascular tissues with a high proportion of parenchyma, mechanically supported by isolated non-vascular fibers. Fibers non-related to the vascular bundles in the culms seem to be a particular feature.

**Figure 5.**
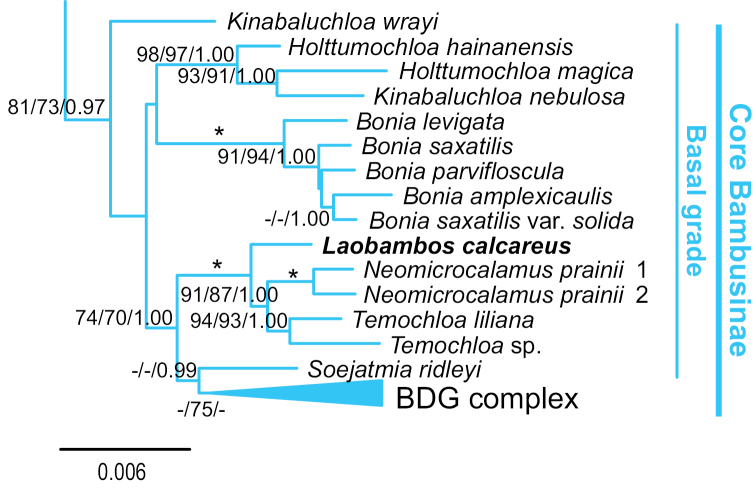
Phylogenetic placement of *Laobambos calcareus*, adapted from Zhou et al. 2017 (the original “Genus indet” has been replaced here by the newly described name).

Molecular analyses (Zhou et al. 2017) demonstrate that *Laobambos* belongs to core Bambusinae, the largest paleotropical woody bamboo subtribe, and is the closest relative to two other (facultatively) karst dwelling taxa; *Laobambos* being the sister group (Fig. 5) of the genera *Temochloa* S.Dransf. + *Neomicrocalamus* Keng f. (represented by *N. prainii* (Gamble) Keng f. in the study), with which it does not share any notable morphological characteristics (*Neomicrocalamus* and *Temochloa* are scrambling bamboos, with hollow to solid culms, culm-leaves apices papery and thin, multiple-branched branch complements with a main dominant branch and sub-equal internodes). *Laobambos calcareus* bears some overall resemblance in habit with another Asian karst dweller, Bonia saxatilis var. solida (C.D.Chu & C.S.Chao) D.Z.Li, to which it is not immediately related (Fig. 5), while also belonging to core Bambusinae (Zhang et al. 2016; Zhou et al. 2017). Bonia saxatilis var. solida, while single-branched, differs significantly from *Laobambos* by culm leaf characters such as the persistence of the culm sheath and the presence of a developed blade, auricles and oral setae.

For woody bamboos, flowering cycles can vary greatly from a couple of years to several decades (up to unknown, in several instances), thus offering no guarantee that this taxon may ever be found flowering in the researchers’ lifetime. The species is undoubtedly new, as a thorough search in the literature of the Indo-Burma biodiversity hotspot area does not mention a bamboo possessing this combination of characters, especially reviewing the literature concerning taxa growing in similar karstic habitats in neighboring countries. The architecture presented by this bamboo is also extremely peculiar (see Fig. 4), and even if the inflorescences, flowers and caryopsis are unknown, we can confidently describe this taxon as new, as no other extant bamboo possesses this combination of characters.

Its generic affinities were clarified when this taxon was placed within a molecular phylogeny (the accession referred to as “Genus indet” in Zhou et al. (2017: fig. 1); the accessions we sequenced resolved as the sister group to two other karst-dwelling taxa: *Neomicrocalamus prainii* (Gamble) Keng f. and *Temochloa liliana* S.Dransf. (Dransfield 2000), with which it shares no morphological characteristics (Fig. 5).

Its morphology is so distinct from other known bamboos that, after consulting several Asian bamboo systematics authorities during the latest symposium on bamboo systematics and ecology (held during the XIX^th^ International Botanical Congress in Shenzhen, RPC, July 2017), they agreed that in such cases there is no reason to further delay its description, due to its singular morphology, even if no fertile material is known.

## Taxonomic treatment

### Laobambos

Taxon classificationPlantaePoalesPoaceae

Haev., Lamxay & D.Z.Li gen. nov.

BA399C4F-318E-5580-AC1A-72AACCC535A0

urn:lsid:ipni.org:names:77211170-1

#### Type species.

*Laobambos calcareus* Haev., Lamxay & D.Z.Li.

### Laobambos calcareus

Taxon classificationPlantaePoalesPoaceae

Haev., Lamxay & D.Z.Li sp. nov.

847DC071-A953-5E24-81F0-ACB2719CC913

urn:lsid:ipni.org:names:77211171-1

#### Type.

Laos • sterile; Khammouane province, Phou Hin Boun, Ban Natan (ບ້ານ​ນາ​ຕານ) area, in bare-rock cracks on slopes of karstic area dolines close to the Canopy Raft camp; 17°59.01'N, 104°48.01'E; elev. 265 m; May 2012; *T. Haevermans*, *V. Lamxay, P. Blanc & F. Hallé, TH852* (holotype HNL!; isotypes: P! (mounted on 4 sheets), K!, KUN!); .

#### Diagnosis.

Similar to Bonia saxatilis var. solida in habit but differs in its succulent culms, persistent culm-sheaths, the unequal structure of its internode pattern, and the absence of culm leaf blade, auricles, or oral setae.

The generic name indicates that the new taxon is restricted to Laos, and the specific epithet emphasizes that the species is restricted to bare karstic crevices.

Shrubby, clumping (non-running) bamboo exclusively growing in karstic rock crevices. Clumps 2–3 m high, 2–3 m in diameter, often with more than 50 culms simultaneously alive per mature clump, with several persistent withered dry old culms; rhizomes pachymorph, short-necked, non-succulent; culms straight, each internode slightly wavy, 6–10 mm in diameter when full of water, cross-section rounded (4–6 mm in diameter when dormant and dry, cross-section star-shaped), solid, and storing water within its mass, deep-green with longitudinal lines of whitish bloom corresponding to the wrinkles (tips of the cross-section star-shape arms) when the bamboo culm shrinks in the dry season; when dried with hot-air for preserving the sample as a herbarium specimen, a thin black wax layer flakes off the culm and greases the paper used for drying; culm leaf, 57 × 12 mm, acute, apical part slightly pubescent but devoid of auricles, cilia or a blade, persisting on culms; no transition between the culm leaf (devoid of blade) and foliage leaf (with inner and outer ligule, pulvinus and fully developed blade). Plant architecture: each culm with a very long first internode, followed by successively shorter and shorter ones (typically 60 cm/40 cm/25 cm/etc., cf. Fig. 4); branch complement always consisting of a single intravaginal branch (Fig. 1D); alternate branching pattern with each branch being roughly as long as the sum of the remaining segments of the main culm, and with the same diameter as the adjacent internode just after the branching (cf. Fig. 4); internode length variation likely dependent on the availability of water during elongation; leaf-complement consisting of around 6–7 non-succulent foliage leaves (Fig. 1B); foliage leaves consistently deciduous during the resting dry season, blades 12–16 mm wide, (50–)80–130(–140) mm long, pseudo-petiole 2–3 mm long with a slightly swollen pulvinus at the base, inner and outer ligules bearing cilia 1 mm long, shoulders asymmetrical, auricles absent. Inflorescences, flowers and caryopsis presently unknown.

#### Other specimens examined.

Laos • sterile; Khammouane province, Haute vallée de la Hin Boun; 16 January 2012; *F. Hallé et al. 4966* (HNL!, P!) • sterile; Khammouane province, Pan a’m, the hill behind Ban Natan, Konglor Cave, 08 April 2013, *T. Zhang et al. 13CS6294* (KUN!).

## Conclusion

DNA sequences comparison informs us that this unique karst-restricted desiccation tolerant bamboo taxon is related to *Neomicrocalamus* and *Temochloa* (Fig. 5). However, no morphological characters unite it to these taxa and thus provides no justification for describing it as a species in one of these two sister genera. We decided to accommodate this species in a new genus to take into account its unique morphology and DNA-based phylogenetic relationships. Further work is needed as no other specimens (fertile or not) have been found in local and international herbaria such as the Paris herbarium (P), which holds a very large representative collection of bamboo specimens from this area. While its formal conservation status cannot be assessed for now due to data deficiency (it is thus tentatively rated Data Deficient), this taxon appears rare and quite restricted in range and habitat as only one population has been located by us or collaborators and thus likely belongs to one of the IUCN threat categories. Extensive fieldwork is required to try to locate other populations of this taxon as well as to collect flowering material if ever possible. Anatomical and developmental studies based on live plants are also necessary to document and understand the water storage cycle in this taxon and determine its abilities and limits in terms of desiccation tolerance. Being a wild relative of economically important tropical bamboos, further research into the adaptations of *Laobambos* toward desiccation tolerance may bring further breeding possibilities and genetic resources for commercial bamboo growers in seasonally dry areas hit by climate change.

## Funding

This research was partly funded by the MAVA Foundation, which funded the 2012 Radeau des Cimes expedition in Laos during which *Laobambos* was discovered. Laos’ authorities made possible the fieldwork and export of the specimens by the Radeau des Cimes expedition organizers according to national and international regulations. Subsequent additional fieldwork funding was provided by the Muséum national d’histoire naturelle PPF grants “Biodiversité actuelle et fossile”, and the Germplasm Bank of Wild Species, Kunming Institute of Botany, Kunming, China. Molecular experiments were supported by a grant of the National Natural Science Foundation of China (no. 31670396). Working visits to China were jointly funded by the French Embassy in Beijing and the Chinese Ministry of Science and Technology (MOST) (TH was the recipient of grants from scientific cooperation programs Xu Guangqi (徐光启) 2012, and “Jeunes Talents France-Chine” 2017) to visit Kunming Institute of Botany and herbarium KUN, as well as South China Botanical Garden Herbarium (IBSC) in Guangzhou for this research.

## Supplementary Material

XML Treatment for Laobambos

XML Treatment for Laobambos calcareus
